# Supplementary material for: Artemisia vulgaris Extract as a Novel Therapeutic Approach for Reversing Diabetic Cardiomyopathy in a Rat Model
Source: Pharmaceuticals (Basel). 2024 Aug 8;17(8):1046. doi: 10.3390/ph17081046 (PMC11358959; doi:10.3390/ph17081046)
Supplement: Supplementary file 1 [file pharmaceuticals-17-01046-s001.zip › pharmaceuticals-3098618-supplementary.pdf]

**Supplementary data**

**1. Body weight and relative organ weight**

Before induction i.e. till 8 weeks the body weight was increased in high fat diet group but after induction the body weight was decreased in Positive Control (PC) group. Standard and *Artemisia vulgaris* treatment restored the body weight towards normal at the end of 16 weeks. The overall relative organ weight was significantly decreased (P≤0.05) in both standard and *Artemisia vulgaris* group as compared to PC as illustrated in supplementary Fig. S1.

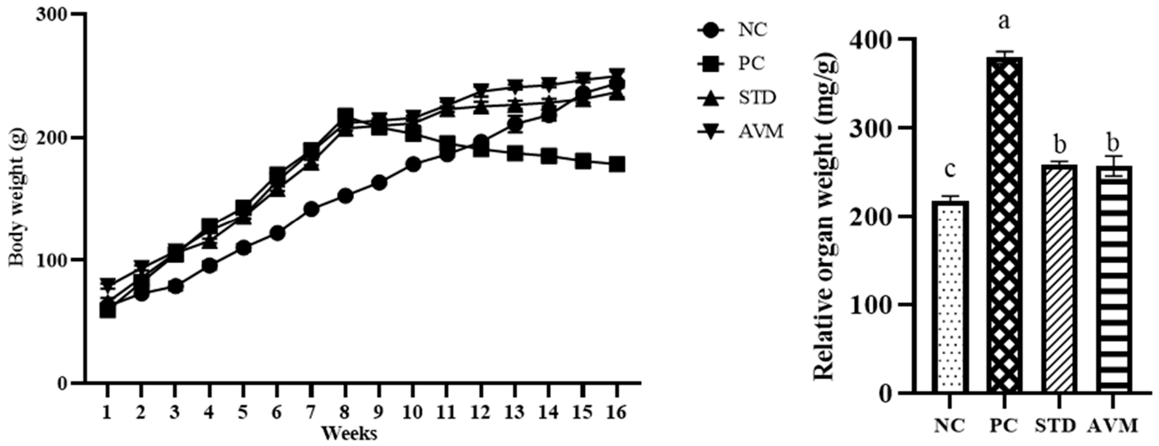


**Figure S1.** Body weight and relative organ weight of negative control (NC), positive control (PC), standard (STD) and *Artemisia vulgaris* methanolic extract (AVM) group. Different alphabets on bars for each parameter show a statistically significant difference (p≤0.05).

**Table S1.** Size of islets of Langerhans of negative control(NC), positive control(PC), standard(STD) and *Artemisia vulgaris* methanolic extract (AVM) group.

| | **NC** | **PC** | **STD** | **AVM** |
|---|---|---|---|---|
| **Diameter (µm)** | 237±6.76 | 108±2.5 | 177±11.26 | 227±10.61 |
| **Area (µm$^2$)** | 744.18±24.47 | 339.12±5.23 | 555.78±35.2 | 712.78±38.12 |
